# The Many Faces of Long Noncoding RNAs in Cancer

**DOI:** 10.1089/ars.2017.7293

**Published:** 2018-09-20

**Authors:** Xue Wu, Oana M. Tudoran, George A. Calin, Mircea Ivan

**Affiliations:** ^1^ Department of Medicine, Indiana University School of Medicine, Indianapolis, Indiana.; ^2^ Department of Microbiology and Immunology, Indiana University School of Medicine, Indianapolis, Indiana.; ^3^ Department of Functional Genomics and Experimental Pathology, The Oncology Institute “Prof. Dr. I. Chiricuta,” Cluj-Napoca, Romania.; ^4^ Department of Experimental Therapeutics, The University of Texas MD Anderson Cancer Center, Houston, Texas.; ^5^ Center for RNA Interference and Non-Coding RNA, The University of Texas MD Anderson Cancer Center, Houston, Texas.

**Keywords:** cancer, non-coding RNA, metabolism, hypoxia, microenvironment

## Abstract

***Significance:*** The emerging connections between an increasing number of long noncoding RNAs (lncRNAs) and oncogenic hallmarks provide a new twist to tumor complexity.

***Recent Advances:*** In the present review, we highlight specific lncRNAs that have been studied in relation to tumorigenesis, either as participants in the neoplastic process or as markers of pathway activity or drug response. These transcripts are typically deregulated by oncogenic or tumor-suppressing signals or respond to microenvironmental conditions such as hypoxia.

***Critical Issues:*** Among these transcripts are lncRNAs sufficiently divergent between mouse and human genomes that may contribute to biological differences between species.

***Future Directions:*** From a translational standpoint, knowledge about primate-specific lncRNAs may help explain the reason behind the failure to reproduce the results from mouse cancer models in human cell-based systems. *Antioxid. Redox Signal.* 29, 922–935.

## Introduction

Since the declaration of War on Cancer in 1971, increased efforts have been focused on characterizing the cellular and molecular changes associated with tumor progression and therapeutic response. Six key hallmarks have been highlighted to describe the mechanisms through which tumor cells proliferate, invade, and metastasize (39), and new emerging hallmarks are added as knowledge advances (40). Nevertheless, despite the progress in understanding the genetic programming of tumor cells, the overall decrease in mortality remains relatively modest. A major reason for therapeutic failure is tumor heterogeneity: advanced tumors being in fact a collection of dynamic subpopulations with different mutation spectra and vulnerabilities. Second, increasingly detailed molecular dissection of tumor-driving signals has revealed additional layers of complexity, with special attention being drawn by the expanding world of noncoding RNAs.

Over the past two decades, these cellular RNAs that translated into proteins have been gradually implicated in virtually all physiological, developmental, and disease processes, including cancer (6, 9, 25, 29, 57, 62). According to the classic dogma, RNA transcripts simply served as templates for protein synthesis (22), which led to decades of protein-centered research. However, successive waves of discovery identified multiple categories of functional noncoding transcripts, beginning with heterogeneous nuclear RNAs (45, 113), followed by introns (4, 5, 20), small nuclear RNAs (37, 38, 63, 81, 102), microRNAs (miRNAs) (61), and long noncoding RNAs (lncRNAs) (10). While the study of miRNAs dominated the first decade of the noncoding RNA revolution, in recent years, lncRNAs—generically defined as noncoding transcripts longer than 200 ribonucleotides—have moved to center stage.

**FIG. 1.**
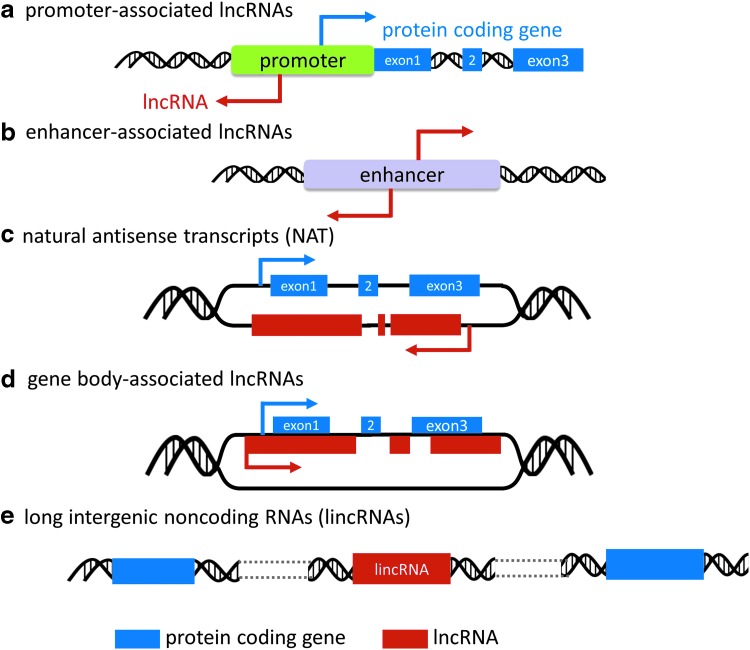
**lncRNA classification.** Based on genomic context, lncRNA can be classified into five categories: **(a)** promoter-associated lncRNAs, **(b)** enhancer-associated lncRNAs, **(c)** natural antisense transcript, **(d)** gene body-associated lncRNAs, and **(e)** intergenic lncRNAs. lncRNA, long noncoding RNA.

The completion of human genome project and the parallel progress in RNA sequencing technology have been instrumental for identification of thousands of lncRNA transcripts (14, 17, 55, 97). In the latest Human GENCODE release (version 26, October 2016, GRCh38, Ensembl 88), 15,787 genes originating 27,720 RNA locus transcripts are identified as lncRNA genes. While the genome size tends to increase during metazoan evolution toward increasingly complex life forms, the number of protein coding genes has remained relatively steady (105). In contrast, the number of noncoding elements, including lncRNAs, appears to have increased dramatically. As the term lncRNA is a generic designation based on size, additional classifications are required when dissecting their biological roles. A popular categorization is based on their genomic contexts (Fig. 1): (i) promoter-associated lncRNAs (Fig. 1a) (*e.g.*, promoter of *CDKN1A* antisense DNA damage-activated RNA); (ii) enhancer-associated lncRNAs (Fig. 1b) (*e.g.*, *Evf2*); (iii) natural antisense transcripts (NATs, Fig. 1c) (*e.g.*, hypoxia-inducible factor 1 alpha antisense 2 [*HIF1A-AS2*]); (iv) gene body-associated (sense) lncRNAs (Fig.1d) (*e.g.*, CCAAT/enhancer binding protein alpha - ecCEBPA); and (v) long intergenic ncRNAs (lincRNAs, Fig. 1e) (*e.g.*, *HOX* transcript antisense RNA [*HOTAIR*], metastasis-associated lung adenocarcinoma transcript 1 [*MALAT1*]) (8, 101).

From a cancer perspective, the ever-increasing number of connections between lncRNAs and oncogenic hallmarks adds a new twist to tumor complexity. lncRNAs tend to be less conserved during evolution and their expression exhibits higher tissue specificity compared with PCGs. Therefore, detailed knowledge about cancer-associated lncRNAs may explain some differences between neoplastic cells derived from different tissues or different species.

Despite the fundamental difference with respect to protein coding ability, lncRNAs exhibit important similarities with PCGs, including chromatin marks at their promoters or enhancers (35). Furthermore, lncRNA genes are also transcribed by RNA polymerase II, spliced at canonical splicing sites, and some undergo polyadenylation (93). Similarly to coding genes, lncRNAs are regulated, positively and negatively, by complexes of transcription factors, coregulators and corepressors, from proximal promoters or enhancers. It is predictable therefore that transcription factors that drive proliferation and survival programs in normal or tumor cells also engage lncRNAs that regulate specific aspects of tumor biology.

In addition to cell-autonomous regulatory mechanisms, the tumor microenvironment has a significant impact in shaping the lncRNA landscape. The combination of stress factors, including oxygen and nutrient depletion, favors the selection of populations with increased ability to survive by rewiring their molecular networks, including metabolism, apoptotic responses, and proliferative programs. As discussed below, it is predictable that tumor microenvironment-regulated lncRNAs should impact this set of basic cell responses.

**FIG. 2.**
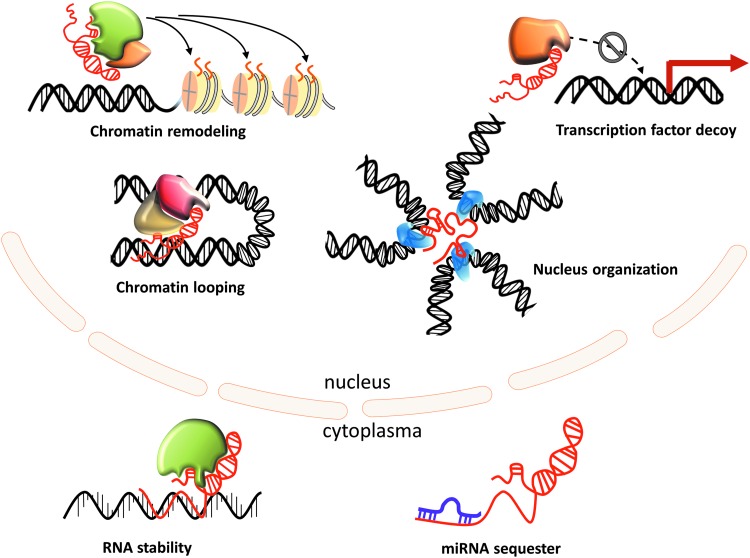
**lncRNA functions.** A diverse range of mechanisms have been described for lncRNA regulation of their targets depending on their subcellular localization: assembly and recruitment of chromatin-modifying complexes to their DNA targets in *cis*; some lncRNAs act as RNA decoys, tethering transcription factors away from their DNA targets by directly binding to them as target mimics; and guiding of the physical looping that occurs between enhancers and targeted promoters (enhancer lncRNAs). Many lncRNAs bind to various protein partners to regulate RNA splicing, degradation, and translation; others act as microRNA target site decoys miRNA, microRNA.

How can a ribonucleotide stretch affect the survival and proliferation of a neoplastic cell? lncRNAs have been shown to regulate gene expression at multiple levels (epigenetic, transcriptional, and post-transcriptional) through interaction with other biomolecules, such as proteins, regulatory DNA regions, and miRNAs (Fig. 2). Subcellular localization appears to be a major determinant for lncRNA interactions and therefore functions. In particular, nuclear lncRNAs modulate gene expression in *cis* or *trans* by interacting with transcriptional coregulators and chromatin remodeling complexes. As shown by Rinn and colleagues, ∼20% lncRNAs associate with the polycomb repressive complex 2 (PRC2), a multicomponent histone methyltransferase required for epigenetic silencing (56). A classic example is provided by *HOTAIR*, shown to reprogram PRC2 and LSD1-CoREST (lysine-specific demethylase 1 and REST corepressor 1 complex) occupancy within the homeobox D cluster (34, 108). Subsequent studies, however, indicated that the lncRNA-based PRC2 guiding model needs to be revised (23, 54). Furthermore, Portoso *et al.'s* recent results suggest that *HOTAIR*-PRC2 interactions are dispensable for *HOTAIR*-mediated transcriptional silencing (90).

A significant proportion of lncRNAs are thought to act in *cis* by enhancing or, conversely, repressing the expression of nearby genes (89). How exactly these lncRNAs perform these functions remains a debated topic. Some of these noncoding transcripts may reorganize the local architecture of chromatin and stoichiometry of transcriptional complexes by specific RNA-protein interactions. Recently, however, Engreitz *et al.* (26) provided a surprising twist to the function of lncRNAs. Their results indicate that (at least in some cases) the lncRNA transcript itself is not critical for the regulation of a neighboring gene as long as there is active transcription of this noncoding locus. In other words, the sequence and interactions of the noncoding RNA product take a backseat to the actual process that generates it. Based on the large number of lncRNAs and the enormous diversity of contexts in which they function, it seems reasonable to assume that these mechanisms are not mutually exclusive.

A puzzling characteristic of lncRNAs is that most of them exhibit very low expression in a particular cell context, including tumors. Many are often considered transcriptional noise and tend to be discounted by arbitrarily set expression cutoff. For lncRNAs, however, low expression should not automatically be viewed as lack of significance as they may achieve biologically meaningful concentrations in specific subcellular compartments; for example, the physical looping mediated by a specific lncRNA bringing together an enhancer and a promoter (66, 89). Supporting evidence has been presented linking lncRNAs to the three-dimensional organization of the nucleus, such as paraspeckle formation or multichromosomal structure (21, 36). These highly specific and localized interactions may support therefore the compatibility between low expression and tissue specificity (84).

At the post-transcriptional level, lncRNAs have been shown to be involved in virtually all steps of RNA metabolism, including stability, processing, and decay. The upregulation of natural antisense (NAT) type of lncRNAs often affects gene expression on the opposite strand by generating RNA duplexes, either by transcript stabilization or degradation *via* RNA interference. Several other effects such as alternative splicing lncRNA-mediated RNA processing have been described as well as an mRNA degradation process called Staufen-mediated decay, which involves lncRNAs binding to the 3′UTR of Staufen-targeted genes (58, 59). Furthermore, lncRNAs can also directly bind proteins, mostly transcription factors, to disrupt their interaction with targeted DNA or other proteins (49).

We will now apply the interactions and functions summarized to the specific case of tumors and highlight how lncRNAs can be integrated in the network of classic neoplastic determinants.

## Tumor Microenvironment and lncRNAs; the Effect of Hypoxia

It is estimated that more than half of solid tumors contain hypoxic regions (75) (11, 110) that represent sources of cells with aggressive phenotype and high resistance to therapy (42, 94, 98, 99). The imbalance between high oxygen consumption of fast proliferating tumor cells and impaired oxygen delivery due to abnormalities in tumor vasculature (30) triggers signaling pathways that regulate tumor cell survival, angiogenesis, metastasis, immune response, and metabolic reprogramming.

Hypoxia-mediated cellular response is primarily driven through the HIF pathway, a complex regulatory network, with multiple feedbacks and checkpoint signaling loops. HIF transcription factors are heterodimers comprising two subunits: an oxygen-sensitive α-subunit (hypoxia-inducible factor 1 alpha [HIF-1α], HIF-2α, and HIF-3α) and a constitutively expressed β-subunit (HIF-1β/ARNT, HIF-2β/ARNT2). While HIF-1β/ARNT is ubiquitously expressed, HIF-2β/ARNT2 is mainly localized in neural tissue and kidney. Three prolyl hydroxylases, EGLN 1–3/PHD 1–3, hydroxylate two proline residues of HIF-α (12, 27, 51), thus favoring the binding of von Hippel-Lindau tumor suppressor protein (pVHL) to HIF-α, which subsequently targets HIF-α for ubiquitination-mediated proteasomal degradation (52, 53, 82). Under hypoxic conditions, the activity of EGLN enzymes decreases, resulting in increased abundance of nonhydroxylated HIF-α subunits, which cannot be recognized by the pVHL complex, thus able to form an active transcriptional complex.

Active HIF regulates the transcription of hundreds of coding and noncoding genes. While a large set of miRNAs have been reported as hypoxia responsive (31), a smaller number of lncRNAs have been identified that respond with at least some consistency to oxygen availability. However, it should not be interpreted that miRNAs are generally more responsive to oxygen deprivation. First, miRNAs have been systematically investigated for a longer period of time (103). Second, multiple nomenclatures coexisted for lncRNAs and one could speculate that the same lncRNA may have been identified by different screens under different names. Finally, omission of nonpolyadenylated transcripts from library preparation may have led to the loss of hypoxia-responsive lncRNAs.

**FIG. 3.**
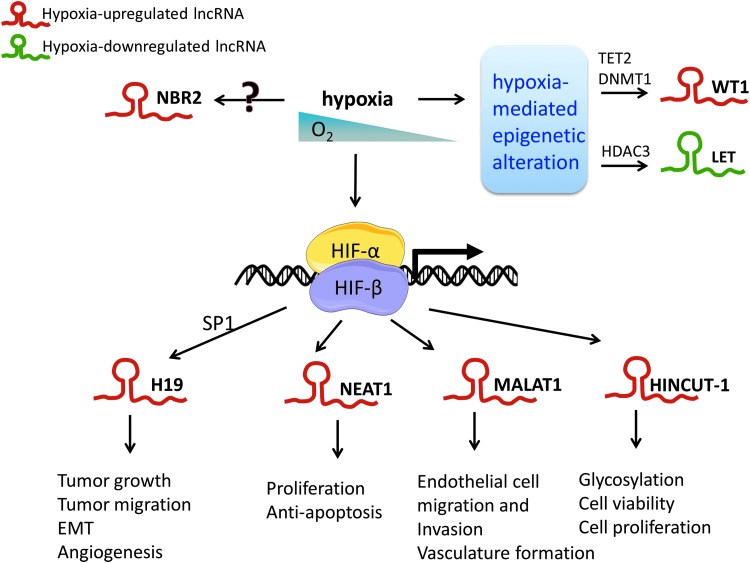
**Examples of hypoxia-regulated lncRNAs.** lncRNAs can be regulated by hypoxia through direct or indirect manner. Some lncRNAs, such as *NEAT1*, *MALAT1*, and *HINCUT-1*, *etc*. have been identified as direct HIF targets. HIF is also involved in upregulation of *H19*, but most likely *via* an indirect signaling through SP1. Hypoxia-mediated epigenetic alterations also play important roles in the expression of some hypoxia-responsive lncRNAs such as *lncRNA WT1* and *LET. H19*, imprinted maternally expressed lncRNA; HIF, hypoxia-inducible factor; *HINCUT-1*, hypoxia-induced noncoding ultraconserved transcript 1; LET, low expression in tumor; *MALAT1*, metastasis-associated lung adenocarcinoma transcript 1; *NEAT1*, nuclear paraspeckle assembly transcript 1; TET2, tet methylcytosine dioxygenase 2; WT1, Wilms' tumor 1.

Overall, two types of lncRNA-HIF connections have been described: lncRNAs that are regulated in response to hypoxia and HIF signaling subsequently and lncRNAs that regulate HIF signaling (Fig. 3). Many hypoxia-inducible lncRNAs reported to date are direct transcriptional HIF targets (18, 19, 28, 79, 85, 86, 121, 124, 130). Chromatin immunoprecipitation sequencing studies of HIF-DNA binding have revealed that ∼30% of HIF binding sites are close to noncoding gene loci; correlations with hypoxic gene regulation revealed significant associations between HIF binding and transcription of lncRNA. Nevertheless, several lncRNAs such as nuclear paraspeckle assembly transcript 1* (NEAT1), MALAT1, HIF1A-AS2*, imprinted maternally expressed lncRNA *(H19)*, hypoxia-induced noncoding ultraconserved transcript 1 *(HINCUT-1)*, and urothelial cancer-associated 1 have been identified as hypoxia-responsive lncRNAs (18, 19, 85, 86). A selection of hypoxia-regulated lncRNAs and their impact on tumor biology are summarized in Table 1.

**Table 1.** Hypoxia-Regulated Long Noncoding RNAs and Their Role in Cancer

*lncRNA*	*Hypoxia response*	*Transcription regulation*	*Cancer type*	*Cancer impact*	*Refs.*
*H19*	Up	HIF/MYC/SP1	HCC	Cell survival and proliferation	(13, 46, 73, 76–80, 87, 117, 122)
			Bladder cancer		
			CRC	Migration	
			Esophageal cancer	Angiogenesis	
			Breast cancer	EMT	
			Gastric cancer		
*HINCUT1/uc.475*	Up	HIF	Colon cancer	Cell proliferation	(28)
*UCA1*/CURD	Up	HIF	Bladder cancer	Cell proliferation	(121)
				Migration and invasion	
				Apoptosis	
*NEAT1*	Up	HIF	Breast cancer	Cell survival	(18)
				Apoptosis	
*MALAT1*	Up	HIF	Breast cancer	Cell cycle	(18, 19, 85, 107)
			Lung cancer	Angiogenesis	
				Tumor metastasis	
*lncRNA-NUTF2P3*-001	Up	HIF	Pancreatic cancer	Proliferation, invasion	(67)
*HIF1A*-AS2	Up	HIF	Glioblastoma	Maintain mesenchymal glioblastoma stem-like cells in hypoxia niches	(7, 86)
			Breast cancer		
*SARCC*	Up	HIF	RCC	Proliferation	(128)
*ANRIL*	Up	HIF	Osteosarcoma	Invasion, apoptosis	(114)
*GAPLINC*	Up	HIF	Gastric cancer	Proliferation	(70)
*PVT1*	Up	HIF	Cervical cancer	Proliferation, apoptosis, migration, invasion, cisplatin cytotoxicity	(47, 50)
			Gastric cancer		
*NBR2*	Up	—	Breast cancer	Cell cycle	(72, 116)
				Apoptosis/autophagy	
				Metabolic checkpoint under energy stress	
*EFNA3 lncRNA*	Up	—	Breast cancer	Tumor metastasis	(32)
*AK058003*	Up	—	Gastric cancer	Modulate DNA methylation at *SNCG* CpG island	(111)
				Migration and invasion	
				Tumor metastasis	
*AK123072*	Up	—	Gastric cancer	Migration, invasion, metastasis	(125)
*linc-ROR*	Up	—	HCC	RNA sponge (*miR145*)	(106)
				Upregulate *HIF1A* mRNA	
				Cell survival	
*HOTAIR*	Up	HIF	Breast cancer	Cell viability	(34, 130)
			Lung cancer	Invasion	
				Apoptosis	
				Metastasis	
*lncRNA-LET*	Down	Hypoxia-mediated epigenetic alteration: HDAC3	Gallbladder cancer	Inhibit invasion and metastasis	(74, 123)
			SCLC		
			HCC		
			CRC		
*WT1 lncRNA*	Up	Hypoxia-mediated epigenetic alteration: DNMT1, TET2	AML	Modulate histone methylation at *WT1* TSS	(83)

AML, acute myeloid leukemia; *ANRIL, CDKN2B* antisense RNA 1; CRC, colorectal cancer; DNMT1, DNA methyltransferase 1; EMT, epithelial-to-mesenchymal transition; H19, imprinted maternally expressed lncRNA; HCC, hepatocellular carcinoma; HIF, hypoxia-inducible factor; *HIF1A-AS2*, hypoxia-inducible factor 1 alpha antisense 2; *HINCUT-1*, hypoxia-induced noncoding ultraconserved transcript 1; *HOTAIR, HOX* transcript antisense RNA; lncRNA, long noncoding RNA; *MALAT1*, metastasis-associated lung adenocarcinoma transcript 1; *NBR2*, neighbor of the *BRCA1* gene 2; *NEAT1*, nuclear paraspeckle assembly transcript 1; *PVT1*, PVT1 plasmacytoma variant translocation 1 lncRNA; RCC, renal cell carcinoma; *SARCC*, suppressing androgen receptor in renal cell carcinoma; SCLC, squamous-cell lung cancer; TET2, tet methylcytosine dioxygenase 2; *UCA1/CUDR*, urothelial cancer-associated 1; WT1, Wilms' tumor 1.

Arguably the first reported hypoxia-inducible noncoding RNA is the transcript generated by the imprinted oncofetal gene *H19* (77, 79, 80). While details remain to be elucidated, Wu *et al.* provided evidence that HIF-1, while required, is not the direct activator of *H19* transcription. They proposed that HIF-1 activates SP1, which in turn activates the *H19* promoter (117). Functionally, *H19* has been shown to promote tumor growth and regulate anchorage-independent growth after hypoxia recovery (77, 79).

*H19* exerts broader proneoplastic effects unrelated to its response to hypoxia. The *H19* gene is highly expressed in common metastatic sites regardless of tumor primary origin. *H19* enhances cell migration *in vitro* and stimulates tumor metastasis *in vivo* (95). In ovarian carcinoma cells, *H19* overexpression is associated with chemoresistance and epithelial-to-mesenchymal transition phenotype (80). *H19* knockdown leads to decreased expression of genes with antiapoptotic function, such as microphthalmia-associated transcription factor, immediate early response 3, protein kinase C, zeta, B cell CLL/lymphoma 3, and serine/threonine kinase 1, and upregulation of proapoptotic genes such as DNA damage-inducible transcript 3 also known as *GADD153* (77). *H19* also plays an important and multipronged role in tumor angiogenesis (77) by regulating the production of proangiogenic factors such as angiogenin, fibroblast growth factor 18, prolylcarboxypeptidase, tumor necrosis factor α-induced protein 1, calponin 2, and inhibitor of DNA binding 2.

Although much remains to be understood about how these complex regulatory effects are set in motion, *H19* actions most likely involve a multitude of interactions, including proteins and RNA interactions. For example, *H19* can modulate chromatin structure within the imprinted gene network through interaction with methyl-CpG-binding domain protein 1 (87). Other studies provided evidence that *H19* functions as an endogenous miRNA sponge for *let-7* tumor suppressor miRNAs (118).

Choudhry *et al.* reported *NEAT1* and *MALAT1* as the main lncRNAs induced in hypoxic MCF7 breast cancer cells (18). *NEAT1* is predominantly controlled by HIF-2, rather than by HIF-1, and is involved in paraspeckle formation (21). One of the paraspeckle functions is to sequester hyperedited RNAs into the nucleus, thus impeding their translocation to the cytoplasm (3). Interestingly, elimination of these lncRNAs in the mouse embryo is compatible with life; therefore, it is conceivable that these transcripts play fine-tuning rather than essential roles in proliferation. However, in tumor cells, hypoxic induction of *NEAT1* promotes proliferation and suppresses apoptosis (18). Protumorigenic roles have been described for *MALAT1* as well. In the highly angiogenic neuroblastomas, upregulation of *MALAT1* promotes endothelial cell migration, invasion, and vasculature formation through fibroblast growth factor 2 upregulation (107). In breast cancer, *MALAT1* regulates critical processes such as tumor growth, differentiation, and metastasis (1). Genetic loss or antisense oligonucleotide (ASO)-mediated knockdown in MMTV-PyMT mouse mammary carcinoma models leads to gene expression alterations and splicing patterns of genes involved in pathogenesis, resulting in reduced branching morphogenesis in MMTV-PyMT- and Her2/neu-amplified tumor organoids, increased cell adhesion, and loss of migration (1). Many miRNAs, including *miR-205*, *miR-200c*, and *miR-204*, have been reported to interact with *MALAT1* and thus contribute to its tumor-promoting mechanism in various cancer types (44, 65, 120). However, these studies often provide little significant molecular proof and require further validation.

HINCUTs are a family of lncRNAs that are transcribed from regions exhibiting extremely high conservation between human, rat, and mouse genomes (2). Our groups have shown that *HINCUT-1* (originally termed *uc.475*) is an lncRNA transcribed as a retained intron of *O*-linked *N*-acetylglucosamine transferase (*OGT*) mRNA and is induced by hypoxia in an HIF-dependent manner (28). Although details are unclear, *HINCUT-1* appears to play an important role in steady-state *OGT* expression and overall cellular glycosylation and its inactivation has detrimental effects on cell viability and proliferation (28).

Recently, suppressing androgen receptor in renal cell carcinoma (*SARCC*) lncRNA was reported as an HIF-2 target in clear cell renal carcinoma. The authors provide preliminary evidence that *lncRNA-SARCC* binds and destabilizes androgen receptor (AR), which results in suppression of AR/HIF-2α/c-MYC signaling (128).

Another lncRNA probably driven by HIF is plasmacytoma variant translocation 1 lncRNA, which appears to be a multifaceted player in cancer. On the one hand, it promotes cell migration and invasion, and on the other hand, it was shown to correlate with immune response stimulation in cervical cancer (50).

Neighbor of breast cancer 1 (*BRCA1*) gene 2 (*NBR2*) lncRNA is a transcript expressed in the opposite orientation from the bidirectional *BRCA1* promoter that has recently been shown to regulate AMP-activated protein kinase under energy stress (72). Wiedmeier *et al.* have recently shown that *NBR2* is induced under prolonged hypoxia in MCF7 cells, while *BRCA1* is repressed. These results suggest that the two transcripts driven by the *BRCA1* promoter are differentially regulated in response to hypoxia, although the regulatory element(s) required for induction of *NBR2* appear to reside outside of the BRCA1 minimal promoter (116).

A rare case of lncRNA reported as downregulated in hypoxia is *lncRNA-LET* (74, 123), which exhibits the behavior of a tumor-suppressing element. In primary hepatocellular carcinoma, *lncRNA-LET* expression is inversely correlated with the prototypical hypoxia marker carbonic anhydrase 9, and experimentally, *lncRNA-LET* downregulation leads to hypoxia-induced cancer cell invasion in hepatocellular carcinoma cells (123). In a different tumor context, ectopic expression of *lncRNA-LET* leads to G0/G1 cell cycle arrest and induction of apoptosis under hypoxic conditions and suppresses gallbladder tumor growth *in vivo* (74).

Occasionally, hypoxia-dependent lncRNA regulation may occur through epigenetic regulators rather than direct HIF activation. In acute myeloid leukemia cells, induction of Wilms' Tumor 1 (WT1) lncRNA, an antisense-oriented lncRNA overlapping with intron 1 CpG island of the *WT1* gene, appears to be the result of demethylation through hypoxia-regulated expression of DNA methyltransferase 1 and tet methylcytosine dioxygenase 2 (83).

**FIG. 4.**
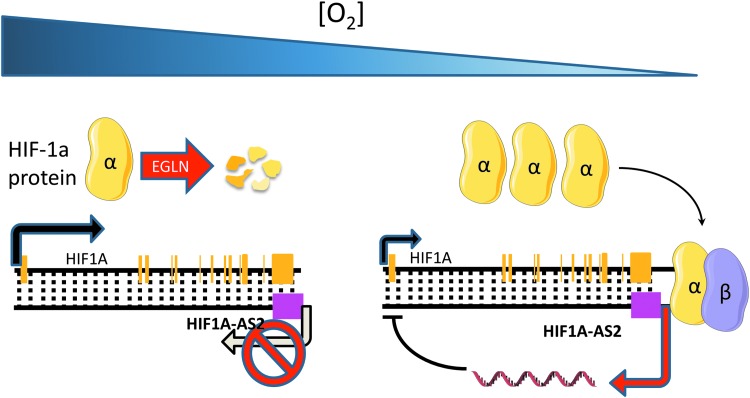
***HIF1A* antisense transcript forms negative feedback loops to control HIF response to hypoxia.** *HIF1A-AS2* is an HIF-target lncRNA. Under hypoxia, HIF directly binds to the *HIF1A-AS2* promoter, driving the expression of an antisense transcript to *HIF1A*, which in return negatively regulates *HIF1A* expression level as a negative feedback mechanism. *HIF1A-AS2*, hypoxia-inducible factor 1 alpha antisense 2.

lncRNAs are not only direct targets of HIF transcriptional activation but also have been demonstrated to regulate the transcription of HIF genes themselves, through direct or indirect interactions. This mechanism creates complex signaling networks with positive and negative feedback loops that integrate multiple signaling pathways to control HIF response to hypoxia. HIF-1α antisense transcripts have long been known to be induced in response to hypoxia (7, 86) and have been shown to negatively regulate HIF expression by chromatin inactivation or mRNA degradation (7) (Fig. 4). More recently, in mesenchymal glioblastoma stem-like cells, *HIF1A-AS2* was found to be the most significantly upregulated lncRNA, playing a protumorigenic role. The authors identified DExH-box helicase 9 and insulin-like growth factor 2-binding protein 2 proteins as major interactors of *HIF1A-AS2* and this interaction in turn drives the expression of tumor-promoting downstream targets, in particular the high-mobility group AT-hook 1 (86). Preliminary evidence suggests that *HIF1A-AS2* may be relevant in a broader context as its knockdown was found to inhibit gastric cancer cell proliferation (16).

A more recent, and incompletely understood, mechanism appears to involve HIF-2 and is based on HIF-2α promoter upstream transcript lncRNA. This is an lncRNA transcribed from the upstream of the HIF-2α promoter, which induces *cis* HIF-2α activation in osteosarcoma (112) and colorectal cancer (126).

**FIG. 5.**
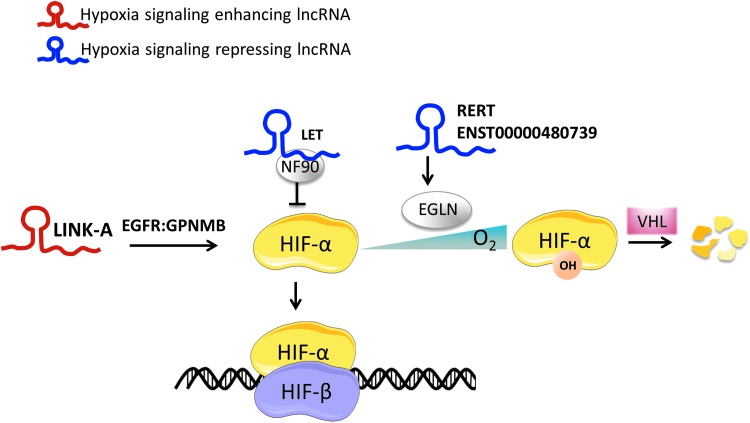
**Examples of lncRNAs that regulate hypoxia signaling.** Several lncRNAs have been reported to regulate hypoxia signaling through indirect mechanisms. *LINK-A* stabilizes HIF-1α through an EGFR:GPNMB heterodimer-dependent HIF-1α phosphorylation and thus activates HIF-1α transcriptional programs. *lncRNA LET* reduces the protein levels of HIF-1α through its association with NF90. *lncRNA RERT* and ENST00000480739 downregulate hypoxia signaling through modulation of EGLN. EGFR, epidermal growth factor receptor; EGLN 1–3/PHD 1–3, prolyl hydroxylases 1–3; GPNMB, glycoprotein nmb; HIF-1α, hypoxia-inducible factor 1 alpha; *LINK-A*, long intergenic noncoding RNA for kinase activation; *RERT*, *RAB4B-EGLN2* read-through lncRNA.

Several lncRNAs have been reported to regulate HIF signaling through indirect mechanisms (Fig. 5). Long intergenic noncoding RNA for kinase activation mediates heparin-binding epidermal growth factor-like growth factor-triggered, epidermal growth factor receptor: glycoprotein nonmetastatic melanoma protein B heterodimer-dependent HIF-1α phosphorylation leading to HIF-1α stabilization, HIF-1α-p300 interaction, and activation of hypoxic programs, including glycolysis under normal oxygen conditions in breast cancer (68). In pancreatic ductal adenocarcinoma, lncRNA ENST00000480739 has been demonstrated to increase the levels of endoplasmic reticulum lectin protein (104), which is known to increase the affinity between HIF-1α and EGLN hydroxylases, therefore leading to HIF-1α destabilization. Somewhat similarly, *RAB4B-EGLN2* read-through lncRNA appears to suppress HIF-1α signaling through *EGLN* transcription activation (132).

## Other Cancer-Associated lncRNAs

Colon cancer-associated transcripts (CCATs), 1 (119) and 2 (69), are lncRNAs transcribed from the highly conserved 8q24 region that has been shown to enhance the transcription of MYC oncogene and promote cancer progression, invasion, and metastasis. *CCAT1* is involved in maintaining chromatin looping between the *MYC* promoter and its enhancers in coordination with the CCCTC-binding factor. *CCAT2* increases chromosomal instability through transcription factor 7-like 2-mediated transcriptional regulation. Thus, both CCAT1 and CCAT2 have been associated with increased risk of cancer and have been shown to regulate multiple molecular pathways to promote cell proliferation, metastasis, and cancer metabolism (96).

*lincRNA-p21* is a p53 transcriptional target that has been shown to repress p53 transcriptional response through heterogeneous nuclear ribonucleoprotein K and trigger apoptosis (48). While in coordination with RNA-binding protein HuR, it inhibits the translation of p53 targets such as jun B proto-oncogene and catenin beta 1 (127). In nonsmall cell lung cancer, tumor samples with high *lincRNA-p21* levels show higher microvascular density. *lincRNA-p21* induces angiogenesis *in vitro*, while lincRNA-p21 inhibition leads to downregulation of angiogenesis-related genes, such as vascular endothelial growth factor A (15).

Prostate cancer-associated ncRNA transcript 1 (*PCAT-1*), although initially reported as a *PCAT* (91), has been described to associate with multiple types of cancers. *PCAT-1* is a target of the PRC2 and represses the transcription of genes involved in cell proliferation, invasion, and metastasis.

*CDKN2B* antisense RNA 1 (*ANRIL*) is transcribed in the opposite direction from the *INK4b-ARF-INK4a* cluster and it is one of the most frequently altered lncRNAs in cancer. The molecular mechanisms through which *ANRIL* mediated cancer development and progression are still uncertain; however, it is hypothesized that aberrant expression levels of *ANRIL* may block the DNA damage response mechanism, leading to genomic instability. In addition, *ANRIL* promotes tumor cell proliferation by regulating target genes in *trans*. *ANRIL* promotes the epigenetically silencing of *miR-99A/miR-449A*, therefore upregulating mechanistic target of rapamycin and cyclin-dependent kinase 6/E2F transcription factor 1 pathways (129).

## lncRNAs: Are Diagnostic and Therapeutic Applications Feasible?

As functional molecules, the lncRNA expression levels may serve as better prognostic and diagnostic indicators of diseases than mRNAs. Additional, their highly specific spatial and temporal expression signatures could lead to a more accurate disease diagnosis and classification. Potential applications of lncRNAs in clinical oncology have been proposed, such as diagnostic biomarkers and therapy response predictors. Prostate cancer-associated 3 (*PCA3/DD3*) lncRNA, for example, has already been tested in controlled clinical settings based on its much higher expression in prostate tumors compared with normal prostate and other tissues. However, based on the available data, *PCA3/DD3* does not appear to be superior to the routinely used prostate-specific antigen (24, 60). Another potentially valuable marker may be *HOTAIR*, which was found to be upregulated dramatically in metastatic breast cancer tissue compared with normal breast tissue (34).

The therapeutic relevance of lncRNAs is currently under exploration, but critical hurdles need to be overcome. Due to their size, transduction of tumor suppressor lncRNAs necessitates delivery systems (*e.g.*, viruses) that have yet to prove their value in clinical settings. On the other hand, oncogenic lncRNAs may be targetable with synthetic RNAs, such as siRNAs, ASOs, or miRNAs. While siRNA-mediated knockdown of cytoplasmic lncRNAs is highly efficient, targeting nuclear lncRNAs is more challenging. Thus, the ASO technology has been optimized to target nuclear lncRNAs for RNase H1-mediated RNA degradation. Promising *in vivo* results have been reported for several lncRNAs such as *MALAT1* (1) and *SAMSSON* (64).

Another approach for lncRNA targeting may be based on lessons learned from the study of vault RNAs (vtRNAs) as mediators of multidrug resistance (33). It was shown that vtRNAs directly bind to chemotherapeutic agents, indicating that it would also be possible to design small molecules that interact with lncRNAs (33). vtRNAs are technically short RNAs, ranging from 80 to 90 nucleotides; however, examples of longer RNAs involved with drug interactions exist, such as aptamers (41, 43, 88, 115). Targeting transcripts the size of lncRNAs may appear challenging, but there is a precedent for fragmenting large ribonucleoprotein complexes into more manageable sizes. This strategy has been applied to design ligands for the expanded rCUG and rCAG repeats expressed in myotonic dystrophy type 1 that interact with Muscleblind-like 1 protein (92). Moreover, unbiased methods such as systematic evolution of ligands by exponential enrichment have the potential to be used to identify molecules that interact with lncRNAs (109).

The clustered regularly interspaced short palindromic repeat (CRISPR)/Cas9-based technologies have revolutionized the study of genetic reprogramming by being developed into a genome-wide editing tool with large applications, including noncoding transcriptome functionality (71, 131). However, CRISPR-Cas9-directed lncRNA genomic deletions do not necessarily induce repression of biological activity (100, 131). The CRISPR interference has been reported as a better approach for lncRNA functionality studies as this technology uses a nuclease-dead dCAS9-KRAB repressor fusion protein to repress gene transcription. This protein can be recruited by single-guided RNA pools to the lncRNA transcriptional start sites, and association with a specific phenotype (71) can be selected based on specific markers as a readout.

In conclusion, multidisciplinary approaches continue to provide critical insights into the involvement of lncRNAs in various aspects of cancer biology. While many lncRNAs already show significant potential as therapeutic targets or cancer biomarkers, transitioning from basic knowledge to viable clinical applications remains challenging. Future studies will need to clarify which, if any, lncRNAs are truly essential for cancer cell viability and to develop more efficient tools for their inactivation in clinical tumors. Furthermore, it would be highly impactful to identify lncRNAs that inform about tumor vulnerability to specific therapeutic agents, potentially in a defined genetic context.

## Abbreviations Used

AML: acute myeloid leukemia
ANRIL: CDKN2B antisense RNA 1
AR: androgen receptor
ASO: antisense oligonucleotide
BRCA1: breast cancer 1
CCATs: colon cancer-associated transcripts
CRC: colorectal cancer
CRISPR: clustered regularly interspaced short palindromic repeat
DNMT1: DNA methyltransferase 1
EGFR: epidermal growth factor receptor
EGLN 1–3/PHD 1–3: prolyl hydroxylases 1–3
EMT: epithelial-to-mesenchymal transition
GPNMB: glycoprotein nmb
H19: imprinted maternally expressed lncRNA
HCC: hepatocellular carcinoma
HIF: hypoxia-inducible factor
HIF-1α: hypoxia-inducible factor 1 alpha
HIF1A-AS2: hypoxia-inducible factor 1 alpha antisense 2
HINCUT-1: hypoxia-induced noncoding ultraconserved transcript 1
HOTAIR: HOX transcript antisense RNA
lincRNA: long intergenic ncRNA
LINK-A: long intergenic noncoding RNA for kinase activation
lncRNA: long noncoding RNA
MALAT1: metastasis-associated lung adenocarcinoma transcript 1
miRNA: microRNA
NAT: natural antisense transcript
NBR2: neighbor of the BRCA1 gene 2
NEAT1: nuclear paraspeckle assembly transcript 1
OGT: *O*-linked *N*-acetylglucosamine transferase
PCA3/DD3: prostate cancer-associated 3
PCAT-1: prostate cancer-associated ncRNA transcript 1
PRC2: polycomb repressive complex 2
pVHL: von Hippel-Lindau tumor suppressor protein
PVT1: PVT1 plasmacytoma variant translocation 1 lncRNA
RCC: renal cell carcinoma
RERT: RAB4B-EGLN2 read-through lncRNA
SARCC: suppressing androgen receptor in renal cell carcinoma
SCLC: squamous-cell lung cancer
TET2: tet methylcytosine dioxygenase 2
UCA1/CUDR: urothelial cancer-associated 1
VEGFA: vascular endothelial growth factor A
vtRNA: vault RNA
WT1: Wilms' tumor 1
